# Spontaneous and therapeutic abortions and the risk of breast cancer among *BRCA *mutation carriers

**DOI:** 10.1186/bcr1387

**Published:** 2006-03-21

**Authors:** Eitan Friedman, Joanne Kotsopoulos, Jan Lubinski, Henry T Lynch, Parviz Ghadirian, Susan L Neuhausen, Claudine Isaacs, Barbara Weber, William D Foulkes, Pal Moller, Barry Rosen, Charmaine Kim-Sing, Ruth Gershoni-Baruch, Peter Ainsworth, Mary Daly, Nadine Tung, Andrea Eisen, Olufunmilayo I Olopade, Beth Karlan, Howard M Saal, Judy E Garber, Gad Rennert, Dawna Gilchrist, Charis Eng, Kenneth Offit, Michael Osborne, Ping Sun, Steven A Narod, the Hereditary Breast Cancer Clinical Study Group

**Affiliations:** 1 The Suzanne Levy Gertner Oncogenetics Unit, The Chaim Sheba Medical Center, Tel-Hashomer, Israel, and the Sackler School of Medicine, Tel-Aviv University, Tel-Aviv, Israel; 2 Centre for Research in Women's Health, Bay Street, Women's College Hospital, University of Toronto, Canada; 3 Department of Nutritional Sciences, University of Toronto, Ontario, Canada; 4 Hereditary Cancer Center, Pomeranian Medical University, Szczecin, Poland; 5 Department of Preventive Medicine and Public Health, Creighton University School of Medicine, Omaha, NE, USA; 6 Epidemiology Research Unit, Research Centre, Centre Hospitalier de l'Universitaire Montréal, CHUM Hôtel Dieu, Département de Nutrition, Faculte du Medicine, Quebec, Canada; 7 Epidemiology Division, Department of Medicine, University of California, Irvine, USA; 8 Lombardi Cancer Center, Georgetown University Medical Center, Washington, USA; 9 Abramson Family Cancer Research Institute, University of Pennsylvania, Philadelphia, PA, USA; 10 Departments of Medicine, Human Genetics, and Oncology, McGill University, Montréal, QC, Canada; 11 Department of Cancer Genetics, Norwegian Radium Hospital, Oslo, Norway; 12 Familial Ovarian Cancer Clinic, Princess Margaret Hospital, Toronto, ON, Canada; 13 British Columbia Cancer Agency, Vancouver, BC, Canada; 14 Institute of Genetics, Rambam Medical Center, Haifa, Israel; 15 London Regional Cancer Centre, London, ON, Canada; 16 Division of Population Science, Fox Chase Cancer Center, Philadelphia, PA, USA; 17 Beth Israel Medical Center, Boston, MA, USA; 18 Toronto-Sunnybrook Regional Cancer Center, Toronto, ON, Canada; 19 Center for Clinical Cancer Genetics, University of Chicago, Chicago, IL, USA; 20 Gynecology Oncology, Cedars Sinai Medical Center, Los Angeles, CA, USA; 21 Hereditary Cancer Program, Division of Human Genetics, Children's Hospital Medical Center, Cincinnati, OH, USA; 22 Dana Farber Cancer Institute, Boston, MA, USA; 23 National Cancer Control Center, Carmel Medical Center, Haifa, Israel; 24 Internal Medicine/Medical Genetics, WCM University of Alberta, Edmonton, AB, Canada; 25 Clinical Cancer Genetics Program, Comprehensive Cancer Center, Division of Human Genetics, Department of Internal Medicine, The Ohio State University, Columbus, OH, USA; 26 Department of Human Genetics and Medicine, Memorial Sloan-Kettering Cancer Center, New York, NY, USA; 27 Strang Cancer Prevention Center, New York, NY, USA

## Abstract

**Introduction:**

*BRCA1 *and *BRCA2 *mutation carriers are at increased risk for developing both breast and ovarian cancer. It has been suggested that carriers of *BRCA1/2 *mutations may also be at increased risk of having recurrent (three or more) miscarriages. Several reproductive factors have been shown to influence the risk of breast cancer in mutation carriers, but the effects of spontaneous and therapeutic abortions on the risk of hereditary breast cancer risk have not been well studied to date.

**Methods:**

In a matched case-control study, the frequencies of spontaneous abortions were compared among 1,878 *BRCA1 *mutation carriers, 950 *BRCA2 *mutation carriers and 657 related non-carrier controls. The rates of spontaneous and therapeutic abortions were compared for carriers with and without breast cancer.

**Results:**

There was no difference in the rate of spontaneous abortions between carriers of *BRCA1 *or *BRCA2 *mutations and non-carriers. The number of spontaneous abortions was not associated with breast cancer risk among *BRCA1 *or *BRCA2 *mutation carriers. However, *BRCA2 *carriers who had two or more therapeutic abortions faced a 64% decrease in the risk of breast cancer (odds ratio = 0.36; 95% confidence interval 0.16–0.83; *p* = 0.02).

**Conclusion:**

Carrying a *BRCA1 *or *BRCA2 *mutation is not a risk factor for spontaneous abortions and spontaneous abortions do not appear to influence the risk of breast cancer in carriers of *BRCA1 *or *BRCA2 *mutations. However, having two or more therapeutic abortions may be associated with a lowered risk of breast cancer among *BRCA2 *carriers.

## Introduction

Germline mutations in *BRCA1 *(MIM # 113705) and *BRCA2 *(MIM # 600185) are estimated to account for about 80% of breast/ovarian cancer families and 20% to 50% of site-specific breast cancer families [1-3]. Mutation carriers face substantially increased risks of developing both breast and ovarian cancer; the lifetime risk for developing breast cancer in *BRCA1/2 *mutation carriers is estimated to be 40% to 85% (4 to 7-fold greater risk than the general population) and for ovarian cancer to be 16% to 64% (a 30-fold increase) [3-5]. Reproductive factors have been shown to modify the risk of breast cancer risk in both mutation carriers [6] and the general population [7,8]. However, the relationships between induced or spontaneous abortions and breast cancer risk in *BRCA1 *or *BRCA2 *carriers have not been well studied. A recent study from Israel suggested that *BRCA1/2 *mutation carriers might be at increased risk for recurrent spontaneous abortions, compared to non-carrier controls [9]; however, this study was relatively small. To date, there has been no published large-scale analysis of the possible effect of being a *BRCA1 *or *BRCA2 *mutation carrier on the rate of spontaneous abortions. The dual aims of this study were: to assess the effect of being a carrier of a germ-line *BRCA1 or BRCA2 *mutation on the risk of spontaneous abortion; and to investigate whether or not there is an association between spontaneous or therapeutic abortions and the risk of breast cancer in women with a *BRCA1 *or *BRCA2 *mutation.

## Materials and methods

### Study population

Eligible study subjects included living women who were identified at one of 55 participating centers in 8 countries. These women were participants in ongoing clinical research protocols at the host institutions. All study subjects (with the exception of those from the University of Utah) received counseling, provided written informed consent for genetic testing and completed a questionnaire that asked for all relevant information regarding family history, reproductive and medical histories, and selected lifestyle factors, including smoking and the use of oral contraceptives. Questionnaires were administered at the individual centers at the time of a clinic appointment or at their home at a later date. Questions addressing fertility history included 'have you ever been pregnant' (yes/no); and the outcome of each pregnancy (for instance, spontaneous abortion, therapeutic abortion, still born, or live born).

The institutional review boards of the host institutions approved the study. In most cases, testing was initially offered to women who had been affected with breast or ovarian cancer. When a *BRCA1 *or *BRCA2 *mutation was identified in a proband or relative, genetic testing was offered to other at-risk women in the family. Mutation detection was performed using a range of techniques, but all nucleotide sequences were confirmed by direct sequencing of DNA. A woman was eligible for the current study when the molecular analysis established that she was a carrier of a deleterious mutation in the *BRCA1 *or *BRCA2 *gene. Most (>95%) of the mutations identified in the study subjects were nonsense mutations, deletions, insertions, or small frameshifts, and clearly considered deleterious and pathogenic.

#### Part 1

The aim of the first part of the study was to examine whether the presence of a *BRCA *mutation influences the rate of spontaneous abortions. Non-carrier controls were women who underwent genetic testing and were found not to be carriers of a deleterious *BRCA1 *or *BRCA2 *mutation. These women were from families where a mutation had previously been identified, who underwent genetic testing at the Centre for Research in Women's Health in Toronto and were found not to carry the family mutation. Since information was not available for European or Israeli controls, we limited this analysis to Canadian and American women (carriers and non-carriers) who had at least one pregnancy and from centers that provided information on all pregnancies, including therapeutic and spontaneous abortions. Potential subjects were excluded if information regarding pregnancy or the number of spontaneous abortions was missing. Women who had experienced ovarian cancer prior to breast cancer were also excluded. After exclusion, a total of 3,503 women were available for this part of the study, including 1,878 *BRCA1 *mutation carriers, 950 *BRCA2 *mutation carriers, 18 women with mutations in both genes, and 657 non-carrier controls.

#### Part 2

Second, we examined whether there is an association between spontaneous or therapeutic abortions and the risk of breast cancer in *BRCA1 *and *BRCA2 *mutation carriers. There was information on cancer history and mutation carrier status for a total of 6,993 women from 55 participating centers who carried a *BRCA1 *or *BRCA2 *mutation. Potential case subjects were selected from among the study subjects with a diagnosis of invasive breast cancer. Control subjects were women who never had breast cancer and who were carriers of a mutation in the *BRCA1 *or *BRCA2 *gene. This study was limited to parous women; therefore, potential subjects were excluded if the women were nulliparous or information on pregnancy was missing (923 women). Not all centers provided data on spontaneous abortions, and data regarding spontaneous abortions was missing for 1,230 women. Potential subjects were excluded if they had been diagnosed with ovarian cancer prior to breast cancer (57 women), if information about ovarian cancer was missing (2 women), if information regarding preventive surgery (bilateral mastectomy or oophorectomy) was missing (109 women), or if information regarding the date of interview was missing (5 women). After exclusions, a total of 4,669 women were eligible, including 2,281 women with breast cancer (potential cases) and 2,388 women without breast cancer (potential controls).

### Statistical analyses

#### Part 1

The Student's *t* test was used to compare the number of spontaneous abortions between carriers and non-carriers. The carrier group was divided into *BRCA1 *and *BRCA2 *carriers. This analysis was restricted to carriers and non-carriers from North America.

#### Part 2

A matched case-control analysis was carried out to test for a possible association between a spontaneous or therapeutic abortion and the risk of breast cancer in *BRCA *mutation carriers. A single *BRCA *mutation carrier (case) was selected for each unaffected subject (control), matched according to mutation in the same gene (*BRCA1 *or *BRCA2*), year of birth (within one year), and country of residence. A diagnosis of ovarian or other form of cancer in the control had to be after the year of diagnosis of the matched case subject. In addition, the date of interview of the controls, age of protective bilateral oophorectomy or age of bilateral mastectomy of the control subject was required to be after the date of breast cancer diagnosis of the matched case subject. A total of 1,694 matched case-control pairs were generated for the analysis, including 1,313 pairs with *BRCA1 *mutations, 380 pairs with *BRCA2 *mutations, and one pair with both mutations. The multivariate odds ratios (ORs), 95% confidence intervals (CIs) and tests for linear trend were estimated by use of conditional logistic regression. A multivariate analysis was carried out to control for the potential confounding effects of age at menarche (years), parity (0, 1, 2, 3, 4+), age of first birth (years), oophorectomy (yes/no), and oral contraceptive use (ever/never). The chi square test was used to test for differences in categorical variables. All statistical tests were two-sided. All analyses were performed using the SAS statistical package, version 9.1.3 (SAS Institute, Cary, NC, USA).

## Results

### Part 1: *BRCA *mutations, parity and spontaneous abortions

There was no significant difference in the number of women who had a spontaneous abortion, or in the mean number of spontaneous abortions, between carriers and non-carriers (Table 1). On average, the non-carriers were born six months after the carriers, so the two groups were similar with respect to age and birth cohort. Because *BRCA1 *and *BRCA2 *mutations are several times more common in the Jewish than non-Jewish population, the analysis was repeated, but restricted to Jewish subjects (Table 2). Three or more spontaneous abortions were experienced by 2.9% of Jewish carriers, 2.3% of non-Jewish carriers and 2.3% of all controls. Among Jewish women, 2.4% of *BRCA1 *carriers, 4.3% of *BRCA2 *carriers and 0% of non-carrier controls experienced three or more abortions. There were significantly more women with three or more miscarriages among the Jewish *BRCA2 *carriers than among the Jewish non-carrier controls (4.3% versus 0%; *p* = 0.03), but these subgroups were small and no Jewish control had 3 or more miscarriages.

### Part 2: Spontaneous and therapeutic abortions and the risk of breast cancer in *BRCA *mutation carriers

The aim of the second part of this study was to examine whether there was an association between the number of abortions (both spontaneous and therapeutic), and the risk of breast cancer among *BRCA *mutation carriers. This analysis was limited to parous women. We compared *BRCA *mutation carriers with and without breast cancer. Case and control subjects were matched for year of birth, mutation status and country of residence (Table 3). They had similar histories of oral contraceptive use, but case subjects had, on average, a significantly earlier age at menarche than the control subjects (12.9 versus 13.0 years; *p* = 0.001) and were less likely to have had an oophorectomy than controls (3.6% versus 6.4%; *p* = 0.0002).

There was no association between the mean number of spontaneous abortions and breast cancer risk in either *BRCA1 *carriers or *BRCA2 *carriers (Table 4). Furthermore, there were no differences in the rate of recurrent (three or more) abortions between cases and controls for either *BRCA1 *carriers or *BRCA2 *carriers (Table 4).

The number of therapeutic abortions was not associated with the risk of breast cancer in *BRCA1 *carriers; however, among women who carried a deleterious *BRCA2 *mutation, ever having had a therapeutic abortion was inversely associated with the risk of breast cancer (Table 5). On average, affected *BRCA2 *carriers had 0.17 therapeutic abortions, versus 0.29 for unaffected *BRCA2 *carriers (*p* = 0.005). Compared to women who never had an abortion, *BRCA2 *mutation carriers who had two or more abortions had a 64% decrease in the risk of breast cancer (OR = 0.36; 95% CI 0.16–0.83; *p* = 0.02).

A protective effect of incomplete pregnancies among *BRCA2 *mutation carriers remained when we assessed the relationship between the total number of spontaneous and therapeutic abortions and breast cancer risk. Among *BRCA2 *mutation carriers, the mean number of spontaneous and therapeutic abortions in total was significantly lower in the cases versus the control subjects (0.69 versus 0.52, *p* = 0.009). Having had two or more spontaneous or therapeutic abortions resulted in a significant reduction in risk (OR = 0.56; 95% CI 0.36–0.89; *p*-trend = 0.009) among women with a *BRCA2 *mutation. The effects of spontaneous and therapeutic abortions, alone and in combination, are presented in Table 6.

## Discussion

Several epidemiological studies have reported that induced or spontaneous abortions are risk factors for breast cancer in the general population [10,11]. However, a collaborative analysis of data from 53 studies, encompassing 83,000 women from 16 countries, concluded that pregnancies that ended in spontaneous or induced abortion did not increase a woman's risk of developing breast cancer [12]. Similar results were obtained subsequently from a study of African-American women [13] and from a prospective study of young women [14]. However, these studies focused on women from the general population, and did not specifically address subgroups of high-risk women. Thus, the results cannot be directly extrapolated to women with a genetic predisposition.

In a previous study of miscarriages among Israeli Jewish women, 4.4% of 343 *BRCA *mutation carriers and 3% of 400 non-carriers reported 3 or more spontaneous abortions [9]. These rates of recurrent abortions are similar to those found in the present study. We also found a higher frequency of recurrent abortions among Jewish *BRCA2 *carriers compared to Jewish non-carrier controls (4.3% versus 0%; *p* = 0.03), but the sample of Jewish controls was small. It is widely held that homozygous carriers of *BRCA1 *or *BRCA2 *germline mutations are non-viable, and it might, therefore, be expected that 25% of conceptions arising from marriages between carriers of mutations in the same gene would end in miscarriage. However, the frequency of marriages between carriers is expected to be low and this phenomenon is unlikely to contribute significantly to the overall miscarriage rate. Furthermore, it is not known at what gestational age the homozygous fetus is likely to miscarry, and if this is an early phenomenon it may be unnoticed. Assuming that 1% of Ashkenazi individuals carry a *BRCA1 *mutation, and if they marry among Ashkenazis at random, then 2.5 per 1,000 of the offspring of carrier women should be homozygous. Given that the proportion of pregnancies that ended in miscarriages among the Jewish women in our study was 15%, or 150 per 1,000, we would expect only 2% of miscarriages among the offspring of carrier women to be due to the excess of homozygote conceptions. The proportion in non-Ashkenazi carriers would be much less.

The results of the second part of the study demonstrate that spontaneous abortions do not influence the risk of breast cancer among women with either a *BRCA1 *or *BRCA2 *mutation. However, ever having had a therapeutic abortion was associated with a 26% reduction in risk among *BRCA2 *mutation carriers. Two or more therapeutic abortions resulted in a 64% decrease in breast cancer risk (OR = 0.36; 95% CI 0.16–0.83; *p* = 0.02). We observed no association between therapeutic abortions and risk among women with a *BRCA1 *mutation (OR for 2 or more therapeutic abortions = 1.00; 95% CI 0.68–1.47). Furthermore, we also found that combined spontaneous and therapeutic abortions conferred protection against *BRCA2 *breast cancers. The results from this study suggest a reduction in the risk of breast cancer in *BRCA2 *mutation carriers with a history of interrupted pregnancies. However, the number of *BRCA2 *mutation carriers was relatively small.

Although ours is a large study, there are several potential limitations that must be considered. Our carrier population is composed of women who seek genetic testing and these women may differ systematically from women in the general population, in terms of demographic features and reproductive histories. For example, it has been shown that women with children are more likely to seek testing than women without children [6]. However, this is likely to be due to a woman's concern for passing down the mutation to her children, and it is not clear that this is also the case for spontaneous or induced abortions.

Secondly, this is a case-control study and women with and without breast cancer were asked to report on their past histories of spontaneous and induced abortions. Women without breast cancer may be more reluctant to disclose a previous induced abortion than women with breast cancer. For this reason, prospective studies are believed to be more reliable than case-control studies for studying the effect of induced abortions. Recently, the Collaborative Group on Hormonal Factors in Breast Cancer [12] published breast cancer relative risk estimates for both spontaneous and induced abortions, derived from multiple studies. The estimates for spontaneous abortion on breast cancer relative risk were 0.98 from both retrospective and prospective studies. However, the estimate of the effect of an induced abortion on breast cancer relative risk was greater for case-control studies (OR = 1.11) than for retrospective studies (OR = 0.93) attesting to the likelihood of recall bias. Both estimates, however, were very close to unity. Furthermore, in our study we observed a protective effect of induced abortions on breast cancer risk in *BRCA2 *(but not in *BRCA1 *carriers) and the direction of the observed effect is opposite to that which we would expect from recall bias.

Various reproductive factors have been shown to exert different effects on the risk of breast cancer among *BRCA1 and BRCA2 *mutation carriers [15,16]. Recently, Cullinane and colleagues [16] reported that increasing parity was a significant risk factor among *BRCA2 *mutation carriers but was protective among *BRCA1 *mutation carriers. The authors found a 50% increased risk of breast cancer among women with a *BRCA2 *mutation who had 2 or more children (OR 1.53; 95% CI = 1.01 to 2.32; *p* = 0.05). These results and those of the present study suggest that the hormonal and/or developmental changes of a short-term pregnancy confer protection against breast cancer in *BRCA2 *mutation carriers, whereas those of a full-term pregnancy will increase the risk of breast cancer. Jernstrom and colleagues [15] reported that breast-feeding was protective among *BRCA1 *mutation carriers but not among *BRCA2 *mutation carriers. The risk factors for *BRCA1 *and *BRCA2 *cancers appear to be different. It is important, therefore, that specific risk profiles be developed for carriers of *BRCA1 *and *BRCA2 *that can be used by genetic counselors when discussing preventive strategies.

## Conclusion

We found that miscarriages and therapeutic abortions do not influence the risk of breast cancer in *BRCA1 *carriers. The possibility of a protective effect of a therapeutic (and possibly spontaneous) abortion among women with a *BRCA2 *mutation requires further investigation.

## Abbreviations

CI = confidence interval; OR = odds ratio.

## Competing interests

The authors declare that they have no competing interests.

## Authors' contributions

Eitan Friedman, conception, planning and drafting of the manuscript; Joanne Kotsopoulos, drafting of the manuscript; Ping Sun, statistical analysis; Steven Narod, coordination of overall research program, drafting of manuscript and final approval; all other authors contributed through the coordination of research activities at their respective institutions.

## Figures and Tables

**Table 1:** Comparison of spontaneous abortions among *BRCA *mutation carriers and non-carriers

	All carriers	*BRCA1*	*BRCA2*	Non-carriers
Age (mean)	49.7	48.8	51.3	46.7
Year of birth (mean)	1950	1951	1948	1951
Number of full-term pregnancies	2.38	2.36	2.42	2.43
Number of spontaneous abortions				
Never	2,103 (73.9%)	1,404 (74.8%)	688 (72.4%)	478 (72.8%)
Ever	743 (26.1%)	474 (25.2%)	262 (27.6%)	179 (27.2%)
1	527 (18.5%)	332 (17.7%)	189 (19.9%)	134 (20.4%)
2	145 (5.1%)	95 (5.0%)	49 (5.2%)	30 (5.0%)
3+	71 (2.5%)	47 (2.5%)	24 (2.5%)	15 (2.3%)
Mean	0.38	0.37	0.40	0.38

Eighteen subjects had a mutation in both *BRCA1 *and *BRCA2*.

**Table 2:** Comparison of spontaneous abortions among Jewish *BRCA *mutation carriers (*n* = 795) and non-carriers (*n* = 110)

	All carriers	*BRCA1*	*BRCA2*	Non-carriers
Age (mean)	51.5	51.1	52.8	47.6
Year of birth (mean)	1947.8	1948.1	1946.8	1950.5
Number of full-term pregnancies	2.15	2.11	2.24	1.94
Number of spontaneous abortions				
Never	595 (74.8%)	436 (75.7%)	154 (73.3%)	78 (70.9%)
Ever	200 (25.2%)	140 (24.3%)	56 (26.7%)	32 (29.1%)
1	140 (17.6%)	100 (17.4%)	36 (17.2%)	27 (24.5%)
2	37 (4.7%)	26 (4.5%)	11 (5.2%)	5 (4.6%)
3+	23 (2.9%)	14 (2.4%)	9 (4.3%)	0 (0.0%)
Mean	0.37	0.34	0.44	0.34

The decimal place (0.0–0.999) following the Year of Birth refers to percentage of 365 days. For example, if a mutation carrier was born on June 30, 1996, this would translate to 1996.5.

**Table 3:** Comparison of case and control subjects with *BRCA1 *and *BRCA2 *mutations^a^

Variable	Case subjects (*N* = 1,694)	Control subjects (*N* = 1,694)	*P* value
Current age (mean)	49.3	48.6	0.22
Year of birth (mean year)	1951.2	1951.8	0.10
Mutation, *N* (%)			
*BRCA1*	1,313 (77.5%)	1,313 (77.5%)	
*BRCA2*	380 (22.4%)	380 (22.4%)	
Country of residence, *N* (%)			
United States	591 (34.9%)	591 (34.9%)	
Canada (excluding Quebec)	491 (29.0%)	491 (29.1%)	
Poland	439 (25.9%)	439 (25.9%)	
Israel	102 (6.0%)	102 (6.0%)	
Norway	55 (3.2%)	55 (3.2%)	
Sweden	7 (0.4%)	7 (0.4%)	
United Kingdom	6 (0.4%)	6 (0.4%)	
Austria	1 (0.1%)	1 (0.1%)	
Italy	2 (0.1%)	2 (0.1%)	
Parity^b^, mean	2.2	2.2	
Oophorectomy	61 (3.6%)	109 (6.4%)	0.0002
Oral contraceptive use, *N* (%)			
Ever	1,002 (59.8%)	1,009 (60.1%)	0.89
Age at menarche, mean	12.9	13.0	0.001

^a^Parity, miscarriages, and therapeutic abortions were censored one year prior to the breast cancer diagnosis of the case. Data for age at menarche, parity and therapeutic abortions are missing for a few study subjects. ^b^Parity includes live born and still born only, and was included in analysis only if birth was one calendar year before the age of diagnosis of the matched case.

**Table 4:** Spontaneous abortions among affected and unaffected *BRCA *mutation carriers

Number of spontaneous abortions	Cases	Controls	OR (95% CI)	*P* value
*BRCA1 *carriers				
Never	1,022	1,032	1	
Ever	291	281	1.09 (0.89–1.32)	0.42
1	210	195	1.14 (0.90–1.43)	0.28
2	53	59	0.94 (0.63–1.42)	0.77
3+	28	27	1.05 (0.61–1.81)	0.86
Mean	0.32	0.31		0.70
				
*BRCA2 *carriers				
Never	295	266	1	
Ever	85	114	0.75 (0.55–1.04)	0.09
1	58	83	0.71 (0.48–1.04)	0.08
2	16	23	0.65 (0.33–1.26)	0.20
3+	11	8	1.54 (0.59–4.01)	0.38
Mean	0.35	0.41		0.30

Odds ratios adjusted for age at menarche (years), parity (0, 1, 2, 3, 4+), age of first birth (years), oral contraceptive use (ever/never) and oophorectomy (ever/never).

**Table 5:** Therapeutic abortions among affected and unaffected *BRCA *mutation carriers

Number of therapeutic abortions	Cases	Controls	OR(95% CI)	*P* value
*BRCA1 *carriers				
Never	1,120	1,109	1	
Ever	192	202	0.98 (0.78–1.22)	0.84
1	137	144	0.97 (0.75–1.26)	0.82
2+	55	58	1.00 (0.68–1.47)	0.99
Mean	0.21	0.22		0.68
				
*BRCA2 *carriers				
Never	329	308	1	
Ever	51	72	0.64 (0.41–1.00)	0.05
1	39	44	0.77 (0.47–1.25)	0.29
2+	12	28	0.36 (0.16–0.83)	0.02
Mean	0.17	0.29		0.005

Odds ratios adjusted for age at menarche (years), parity (0, 1, 2, 3, 4+), age of first birth (years), oral contraceptive use (ever/never) and oophorectomy (ever/never).

**Table 6:** Therapeutic and spontaneous abortions among *BRCA1 *and *BRCA2 *mutations carriers

	Cases	Controls	OR (95% CI)	*P* value
*BRCA1 *carriers				
Neither	887	888	1	
Therapeutic only	135	144	0.98 (0.74–1.26)	0.82
Spontaneous only	233	221	1.09 (0.87–1.35)	0.46
Either	368	365	1.04 (0.87–1.59)	0.69
Both	57	58	1.06 (0.71–1.59)	0.78
				
*BRCA2 *carriers				
Neither	253	216	1	
Therapeutic only	42	50	0.68 (0.42–1.11)	0.12
Spontaneous only	76	92	0.78 (0.55–1.11)	0.16
Either	118	142	0.75 (0.55–1.02)	0.06
Both	9	22	0.36 (0.14–0.88)	0.02

Odds ratios adjusted for age at menarche, parity, age of first birth, oral contraceptive use (ever/never) and oophorectomy (ever/never).
